# Consolidation, Stages of Change, and Loyalty among Users of Public Sports and Health Services Aged 12–16

**DOI:** 10.3390/ijerph181910113

**Published:** 2021-09-26

**Authors:** Antonio Fernández-Martínez, Luis Alberto Dueñas-Dorado, María Rosario Teva-Villén, Alberto Nuviala

**Affiliations:** 1 Department of Sports and Computer Science, Pablo de Olavide University, Crta. de Utrera, Km1, 41013 Seville, Spain; rteva@upo.es; 2 Faculty of Sports Organisation, Universidad Autónoma de Nuevo Leon (Mexico), Cd. Universitaria, s/n, San Nicolás de los Garza 66455, N.L., Mexico; luis.duenasdrd@uanl.edu.mx

**Keywords:** perceived quality, satisfaction, loyalty, stages of change, adolescents

## Abstract

There are two main lines of inquiry in the literature on adherence and/or loyalty to the practice of physical activity and to health services: one focuses on the impact of perceived quality of sports and health services and satisfaction with these services on user loyalty, while the other concludes that users with more self-determined motivation at more advanced stages of physical activity display higher levels of physical activity and greater intentions to continue this activity. The objective of this study is to ascertain the impact of different dimensions of sports service quality on satisfaction and loyalty among users aged 12 to 16 years old and to identify any differences between adolescent users at more and less consolidated stages of physical activity. A total of 1717 minors with a mean age of 13.83 ± 1.32 years who practise organised physical activity at public sports centres in Nuevo León (Mexico), 51.5% of whom were boys, participated in the study. The model of structural equations linking quality, satisfaction, and loyalty displayed adequate indices. The results showed that the staff, specific activity, and user satisfaction are predictors of loyalty. Significant differences were only found between minors at consolidated and non-consolidated stages of physical activity in the relationship between service personnel and loyalty. In conclusion, human resources and their deployment are predictive of loyalty towards sports and health services among adolescents.

## 1. Introduction

Understanding users of sports and health services is a useful strategy for maintaining and increasing levels of physical activity among the population [1,2]. The literature has shown that advanced stages of physical activity are linked to high levels of this activity [3] and greater willingness to do exercise [4]. Meanwhile, other studies associate high levels of physical activity with satisfaction with the service received and greater loyalty to the organisation [5]. However, no research has been carried out so far into the impact of stages of change on the relationship between quality, satisfaction, and loyalty to sports services in adults or in children and adolescents, despite the importance of habits established during childhood for future behaviour in adult life [6]. Physical activity in children has been proven to determine their current health, future health, and lifestyle throughout adolescence and adulthood [7]. Therefore, this study aims to explore the dimensions of quality that influence satisfaction and loyalty among adolescent users of sports services and to ascertain the impact of stages of behaviour change on the relationships between these concepts.

Sedentary behaviour and a lack of physical activity have risen [8] to become one of the main causes of mortality around the world [9]. Inactivity and sedentary behaviour are currently a cause for concern for the public health authorities [10,11] due to their significant influence on mortality and cardiometabolic morbidity, as well as on the appearance of certain cancers [12]. High levels of sedentarism have been observed among children and adolescents, 80% of whom are inactive and fail to meet recommendations for physical activity [13,14]. These sedentary behaviours among children and adolescents are associated with early metabolic and cardiovascular risk [15,16]. Behaviour in childhood has been shown to directly influence behaviour in adulthood [17]. As a result, physical activity and sedentarism among children determine their current health and are a clear predictor of their health and lifestyle during adolescence and adulthood [7].

**Figure 1:**
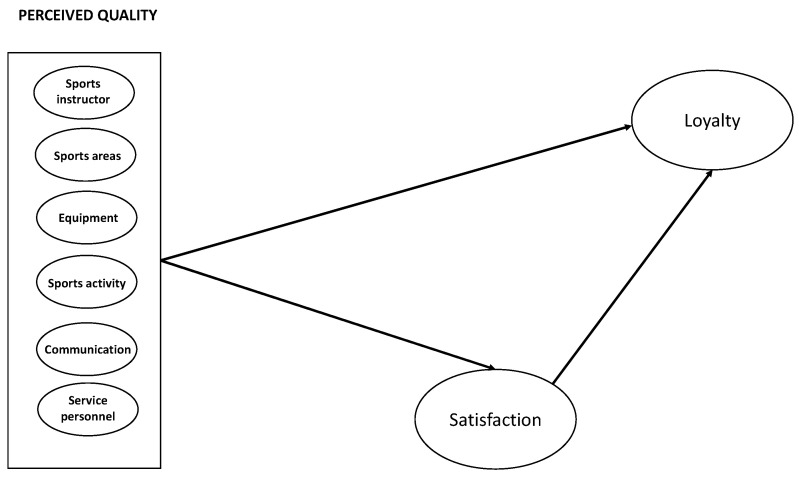
Structural model predicting loyalty to sports and health services among users aged 12–16.

Recent studies have demonstrated the relationship between quality, satisfaction, and loyalty to the sports organisation among adult users of sports services [2,18] and even among adolescent users [19] as a strategy for encouraging adherence to and maintenance of physical activity levels. According to Oliver [20], loyalty is understood as a strong commitment to purchase goods or services on an ongoing basis into the future, despite the possibility of competitors’ efforts and/or situational influences bringing about a change in behaviour. The concept of loyalty is related to service continuity [21]. In other words, consumers who repeatedly return to the same service are loyal [22], but building loyalty and encouraging repeat visits in the sports and health services market also entails the acquisition of healthy habits [23]. Most of the studies that have analysed the relationship between these concepts have used unidimensional models with adults [18,24,25]. Only Pérez-Órdas et al. [19] used a model in adolescents to analyse relationships between the different dimensions of quality and loyalty. Unfortunately, their study did not consider the relationships between dimensions of quality and satisfaction or potential indirect relationships with loyalty via satisfaction. A lack of information prevents programme managers from developing reliable strategies with an objective impact on sports and health services. This shortcoming may be due to the failure to explore secondary concepts, as the primary objective of most studies is to determine the role of service quality in its relationships with other variables [23] rather than to ascertain the importance of the model’s dimensions. The order of the structural model in Figure 1 should start from the left with Perceived Quality (independent variable) and end with Satisfaction and Loyalty (dependent variable). Also, the structural model should include different dimensions of the quality of sports services in Figure 1.

In order to understand adherence to and maintenance of sports practice, it is also necessary to identify the influence of motivation [26]. Mechanisms for adherence to different physical exercise programmes are highly diverse [27] and an analysis of these mechanisms is key to understanding people’s commitment to sport [28]. The influence of motivational processes on the practice of and adherence to physical or sports activities has been amply studied [3] from two main independent theoretical perspectives: the transtheoretical model (TTM) of behaviour change [29] and self-determination theory (SDT) [30,31]. TTM comprises stages and processes of change. The stages explain when and the processes explain how changes in individuals’ attitudes, intentions, and behaviour come about [32]. The model posits the idea that physical exercise is a dynamic behaviour and that people go through five stages of behaviour change in their attempts to exchange their sedentary behaviours for a more physically active lifestyle [3]. The stages included in the model are: pre-contemplation (the subject does not engage in physical activity and has no intention of doing so), contemplation (the subject is inactive but intends to change), preparation (the subject is active but does not fulfil the recommendations for healthy practice), action (the subject is active and fulfils the recommendations for healthy practice but has not yet completed six full months of regular practice) and maintenance (the subject has engaged in healthy physical activity for more than six months). The action and maintenance stages of behaviour change are the most active [3,33].

SDT [34] is the most commonly used motivational theory in the study of physical exercise. It is used to explain the phenomenon of adherence to sports practice [35]. In SDT, motivation is situated on a continuum where three levels may be identified [36,37]: intrinsic motivation (the most self-determined: engaging in an activity out of personal pleasure), extrinsic motivation (engaging in an activity for external reward or recognition), and amotivation (the least self-determined). Three basic psychological needs (BPNs) are established: autonomy (engaging in activities on one’s own initiative), competence (interacting efficiently with one’s surroundings to feel competent) and relatedness to others (feeling part of a group) [31,34]. In SDT, BPNs are psychological mediators that influence the three main types of motivation [30,31]. Studies have shown that high levels of self-determined motivation act as a predictor of physical activity [38,39]. Other research has revealed that highly motivated individuals dedicate more time to sport and engage in sports practice more frequently [40]. Scientific evidence has also demonstrated the positive association between self-determined motivation and levels of physical activity during leisure time among adolescents [41].

Some studies have established a relationship between the stages of change and motivation, or between SDT and TTM. Their findings show that people at the action and maintenance stages of change appear to experience more self-determined motivation [3,42,43,44]. It has even been observed that people at more advanced stages display greater willingness to engage in exercise [4]. Two main lines of inquiry may be identified in the literature. One points to the influence of quality and satisfaction on user loyalty (understood as a greater frequency of sports practice) and the presence of higher levels of physical activity among users who are loyal to sports organisations. The other concludes that users with more self-determined motivation at more advanced stages of physical activity display high levels of physical activity and express a stronger intention to continue with it. Therefore, it is relevant to explore how different dimensions of the quality of sports services influence satisfaction and loyalty among users aged 12 to 16 and to identify any differences between minors at the maintenance stage and at the action stage. If such differences are found to be present, different strategies for encouraging adherence to sports and health practice would be required to reflect the needs of minors at each stage (Figure 1).

## 2. Materials and Methods

### 2.1. Subjects

The study population was made up of 1717 minors with a mean age of 13.83 ± 1.32 who engage in organised physical activity at public sports centres in the metropolitan area of Monterrey, Nuevo León (Mexico). Boys slightly outnumbered girls in the sample (51.5%). The average session duration was 104.97 ± 55.48 min, and 14.9% of users completed fewer than three sessions of physical activity per week. 42.3% claimed to engage in physical activity four or more times per week. 16.6% of the users were at less consolidated stages of physical activity (precontemplation; contemplation; preparation).

### 2.2. Instruments

To identify the stage of change occupied by users of municipal public sports services, Marcus & Forsyth’s questionnaire was used [32]. The questionnaire was adapted and validated for the Mexican context by Zamarripa, Ruiz-Juan, & Ruiz-Risueño [3]. It consists of a brief definition of the concepts of physical activity and regular physical activity, as well as four statements requiring a dichotomous response (yes/no): (1) I am currently physically active; (2) I intend to become more physically active in the next 6 months, (3) I currently engage in regular physical activity; and (4) I have been regularly physically active for the past 6 months. Prior research has confirmed the questionnaire’s reliability and criterion validity [3,32].

The quality of the services and user satisfaction with them were assessed using the 24 items of the EPOD2: Sports Organisations Perception Scale, version 2 [25]. The items are grouped into seven dimensions: sports instructors, service personnel, equipment, sports facilities, communication, activity, and satisfaction. The instrument is a Likert scale ranging from 1 (strongly disagree) to 5 (strongly agree). Loyalty was measured via four items on a scale of future intentions among sports service users that ranged from 1 to 7 [45] (Table 1).

### 2.3. Procedure and Research Design

Before data collection began, a letter was sent to the directors of the municipal sports centres participating in the study to request permission to proceed (San Nicolás de los Garza, Monterrey, Escobedo, and Guadalupe). The participants’ legal guardians were then asked to consent to their participation in the study. Participation was anonymous and voluntary and the participants were able to ask questions about any items they did not fully understand. All participants were informed of the security measures taken to protect their anonymity and image and ensure the confidentiality of all data, and were told that there were no right or wrong answers. They were asked to respond sincerely and honestly.

Participants took approximately 10 min to fill out the questionnaires. The questionnaires were administered at the reception of the municipal sports centres, where a person was appointed to supervise their completion and answer any questions that arose. This study complies with international ethical guidelines as recommended by the American Psychological Association. Ethical approval was obtained from the Universidad Autónoma de Nuevo León (Mexico), review committee 16CI19039021. The study was also approved by the Vice-Rectorate for Research and Technology Transfer at the Universidad Pablo de Olavide in Seville.

### 2.4. Statistical Analysis

First, a series of exploratory tests of the items were carried out, such as the calculation of frequencies, means, standard deviations (SD), skewness, kurtosis and factor loading. In order to verify the reliability and validity of the instruments used in this research, correlations between the study constructs, average variance extracted (AVE), composite reliability (CR), and Cronbach’s alpha were calculated. Analysis of variance (ANOVA) and contingency tables were used to compare means and proportions between groups of the latent variables studied, studying the size of the effect. Calculations were performed using a spreadsheet in Excel and the Statistical Package for the Social Sciences (SPSS, IBM, Armonk, NY, USA), version 22.0. The model linking perceived quality (sports instructors, service personnel, equipment, sports facilities, communication, activity), satisfaction, and loyalty was then tested using the programme Analysis of Moment Structure (AMOS, IBM, Armonk, NY, USA), version 22.0. A confirmatory factor analysis of the model was carried out, followed by a multi-group analysis. Byrne’s guidelines [46] were followed: firstly, the model fit for each sample was checked separately (total population, model 0; users at less consolidated stages, model 0a; users at consolidated stages, model 0b). The variation of the model between the groups was then checked; this involved specifying a model with equal parameters for all groups and comparing this model with a less restrictive model with parameters free to take any value. This procedure allowed the invariance of the factor structure of the model to be checked. The aim of the analysis was to determine whether the model linking quality and its dimensions to satisfaction and loyalty was the same for both groups of users: users at less consolidated stages and users at consolidated stages of physical activity. The maximum likelihood method was used [47]. The adjustment of each model was assessed by examining various indices. The Root Mean Square Error of Approximation (RMSEA), the Comparative Fit Index (CFI), the Expected Cross-Validation Index (ECVI), and the Akaike Information Criterion (AIC) were used. The *χ*^2^ value (CMIN) and the *χ*^2^ value/degrees of freedom (CMIN/DF) were also used. RMSEA values < 0.07 indicate an acceptable fit [48] and RMSEA values ≤ 0.06 indicate a good fit [49]. CFI values ≥ 0.95 are considered to be acceptable [49]. Small AIC and ECVI values suggest a good model fit [50]. With respect to the *χ*^2^ value/degrees of freedom ratio, a perfect model would yield a value of 1.00, and ratios below 2.00 would be considered to be indicators of a very good model fit, while values below 5.00 would be considered to be acceptable [49,51,52]. Invariance in measurement between groups was assessed using the Δ*χ*^2^ test and the recommendations of Chen [53] and Cheung & Rensvold [54] were followed, which state that the cut-off values ΔCFI ≤ 0.01 and ΔRMSEA ≤ 0.015 indicate the absence of differences between models. Finally, the standardised regression coefficients were calculated for the relationships in the model by groups of users.

## 3. Results

83.4% of the minors in the study were at advanced stages of consolidation of physical activity. The majority of the participants were at the maintenance stage. Gender differences were observed between the different stages. A higher proportion of girls were at consolidated stages. No significant differences were observed by age. An analysis of the dimensions of quality revealed significant differences between the consolidated group and the less consolidated group for all dimensions. The consolidated group rated all dimensions of service quality higher than users at less consolidated stages; the same phenomenon was observed for satisfaction and loyalty (Table 2).

Once the presence of differences in quality assessments, satisfaction, and loyalty to the sports organisations providing services to the minors had been confirmed, it was time to identify any differences in the relationships between the different dimensions and the factors comprising the model that links quality, satisfaction, and loyalty. Firstly, the model was tested. Table 3 shows that the analysed model displays adequate adjustment indices for the total study population (model 0), for users at less consolidated stages (model 0a), and for users at consolidated stages (model 0b).

In order to compare the model for the two groups of minors classified by stage of change (Table 2), factor invariance tests were performed. When the difference in *χ*^2^ between the unrestricted models (model 1) and the other models was considered for the two groups of minors, significant differences became apparent. There were also differences when models 2, 3, 4, and 5 were compared with one another. Meanwhile, the CFI and RMSEA values for all the models were very similar, with a difference between them of less than 0.01 and 0.015 respectively, indicating the factor invariance of the model for studying differences by minors’ stages of change (Table 3).

The results have shown that sports instructors, service personnel, and the specific activity are predictors of satisfaction for the total study population. Satisfaction and the activity factor have a direct effect on loyalty, while sports instructors, service personnel, and activity are indirect predictors of loyalty. These results are repeated in the population at a consolidated stage of physical activity. It is important to note that communication is a predictor of loyalty among consolidated users but not among the total population. However, service personnel and activity are predictors of satisfaction among the group of less consolidated users. Only service personnel have a direct effect on loyalty among minors at less advanced stages of consolidation of physical and sports practice (Table 4).

## 4. Discussion

Sedentary habits and a lack of physical activity have risen among the general population, becoming a significant concern due to the risks of mortality and morbidity that they entail. Sedentary behaviours are replicated by children and adolescents, resulting in a decline in their current health and a direct impact on their future health and lifestyle. Therefore, it is important to maintain and increase levels of physical activity among children and young people as high levels of physical and sports practice are associated with consolidated stages of physical activity and greater willingness to exercise. Sports and health services must seek strategies to encourage the practice of physical activity and sport among their users, especially among children. The aim of this study was to ascertain which dimensions of the quality of sports and health services influence satisfaction and loyalty among users aged 12–16 and how these dimensions affect the relationships between these concepts and the stages of behaviour change.

To confirm the validity and reliability of the study, the items were first analysed quantitatively. The mean score of the study items and factors was examined. The results showed values around the middle of the scale and a standard deviation close to 1, demonstrating the normality of the results according to Carretero-Dios & Pérez [55]. Reliability was confirmed using Cronbach’s α, obtaining adequate values. Convergent validity was determined via correlations between the study factors using Pearson’s coefficient. The correlations were positive and significantly related, demonstrating this type of validity. Another test used to ascertain the instrument’s validity were the CR and AVE values. The acceptable values were >0.6 for CR and >0.5 for AVE [56,57]; the results exceeded the values suggested in the literature, so they were confirmed to be valid.

The validity of the model linking quality and its dimensions to satisfaction and loyalty was then tested via a confirmatory factor analysis. The parameters were estimated using the maximum likelihood method, following Thompson’s recommendation [47]. To evaluate the adequacy of the model, a group of indices were assessed jointly (CMIN, DF, CMIN/DF, CFI, RMSEA, AIC, and ECVI). Byrne’s [46] suggestions were followed, so the model was studied for the total population (model 0) and for the two groups of users: minors at less consolidated stages (model 0a) and minors at advanced stages (model 0b). The fit indices were adequate for all models. The variance of the model between the groups was then checked; this involved specifying a model with equal parameters for all groups and comparing this model with a less restrictive model with parameters free to take any value. This procedure allowed the invariance of the factor structure of the model to be checked. When the difference in *χ*^2^ between the unrestricted models (model 1) and the other models was considered for the two groups of minors, significant differences emerged. There were also differences when models 2, 3, 4, and 5 were compared with one another. Since CMIN/DF is sensitive to sample size, the criterion established by Cheung & Rensvold [58] with regard to the ΔCFI and the ΔRMSEA was also used. According to them, invariance is admitted when ΔCFI ≤ 0.01 and ΔRMSEA ≤ 0.015. The CFI and RMSEA values for all the models were very similar, with a difference between them of less than 0.01 and 0.015 respectively, indicating the factor invariance of the model for studying differences by minors’ stages of change.

The descriptive analysis of the results shows that most minors using sports and health services are at the maintenance stage, which appears logical as this population regularly engages in physical activity. Moreover, according to Daley & Duda [58], who view the TTM in quantitative terms, there is a relationship between the amount of physical activity practised and the more advanced stages. In order to be included in the maintenance stage, users had to have been active throughout the past six months. This points to the considerable levels of physical activity practised by the majority of users of these types of services. Another aspect of note are the significant differences in the percentage of users at more advanced stages of consolidation of physical activity, with a higher proportion of girls found at these stages. The researchers had already observed this difference among university students, attributing it to greater self-determined motivation among women. Future research could explore how motivation affects quality, satisfaction, and loyalty to sports and health services.

The multi-group analysis showed that satisfaction is predicted by sports instructors, service and administrative personnel, and the specific activity in the total population and in the group of minors at more consolidated stages of physical activity. Despite the absence of differences between the groups of minors, only the activity dimension and the service and administrative personnel dimensions were predictors of satisfaction among the minors at less consolidated stages. This difference may owe to motivational issues, as Nuviala et al. [26] demonstrated in a study grouping users of these services by their motivations for practising physical activity. Higher levels of self-determined motivation are found among groups at more consolidated stages of physical activity, as Dueñas-Dorado et al. and Zamarripa et al. [3,5] observe. Equally, it is important to note the similarity between the results of this study and the findings of Haro-González et al. [59] in elderly women using sports services. The sports facilities dimension was found not to predict loyalty; nor did being a woman who attends sports centres not exclusively catering to women. This finding was explained by the different socioeconomic profile of the two groups of women. Therefore, motivation and the socioeconomic profile of sports and health service users could be two important variables to consider when implementing strategies to improve quality and build loyalty.

A direct relationship between satisfaction and loyalty was also observed among the total population and the minors at consolidated stages of physical activity, demonstrating the significant role of satisfaction in building loyalty [18,60,61], one of the most important factors influencing users’ future intentions and behaviours. It is important for these services to achieve high levels of satisfaction [19] as building loyalty in the sports and health services market leads to the development of more healthy lifestyles [23].

With regard to the relationships between the dimensions of quality and their effect on loyalty to sports and health services, the activity dimension emerged as the only predictor of loyalty for the total population. Nuviala et al. [62] showed that the programme of activities was a key component in school-age children’s participation, as there is a direct relationship between activity quality and level of practice. Quality in programmes of sports activities is understood as a range of services that reflects minors’ interests and needs as closely as possible, encouraging greater loyalty among them [63]. Therefore, it is important to determine adolescents’ motivations for engaging in sports activities [64]. Sports instructors, service and administrative personnel, and the specific activity have an indirect relationship with loyalty via satisfaction in the total population. These results can be observed in the group of minors at consolidated stages of practice.

Interestingly, communication is a direct predictor of loyalty in the group of minors at more consolidated stages. Fernández-Martínez et al. [65] demonstrated the presence of this direct relationship in sports and health services among Spanish minors of both sexes. Despite the effect of communication on loyalty, this topic has not been studied in depth and few studies have sought to examine it. The importance of word-of-mouth [45,61] as a vehicle for influencing peers to engage in the specific behaviour of physical activity [66,67] has also been overlooked.

Another of the most relevant findings is that the only element to display significant differences between the groups is the direct influence of service and administrative personnel on loyalty towards sports and health services among minors at less consolidated stages, whereas this influence is indirect among the total population and the group at more consolidated stages. This is a hugely important finding given the role played by this group of employees. Haro-González et al. [59] and Macintosh & Doherty [68] highlighted the importance of these personnel for user satisfaction. It is possible that the influence of service and administrative personnel originates in the organisational culture, which influences customer perceptions [68] and user satisfaction [69]. For this reason, sports and health services should work on organisational culture with their staff as a key variable, especially service and administrative personnel who add value to the range of activities on offer [63].

Due to the importance of consolidating physical activity among young people (as it results in improved current and future health and consolidates the habit of sports practice and healthy activities), it is important to continue researching this topic and introducing new elements or modifying existing ones. It would be interesting to conduct a more detailed analysis of the sociodemographic profile of service users and assess the impact of this variable on loyalty. Moreover, high levels of self-determined motivation are associated with high levels of adherence to sports practice, but whether or not intrinsic motivation directly modifies the relationship between quality, satisfaction, and loyalty remains unknown.

Human resources (sports instructors and service personnel) have become one of the key components of loyalty towards sports and health services. It would be helpful to analyse the profile of these professionals in order to find out more about them. Communication from the organisation, be it directly via its human resources or indirectly via different types of messages and channels, is another relevant dimension to consider. Assessing the influence of communication on loyalty to the service and adherence to the practice of physical activity would be another fruitful line of research.

The activities themselves, another key component of adherence, should be analysed in more detail. It would also be interesting to find out whether or not the use of different methods by sports instructors affects adherence to physical activity. This relationship may differ depending on the type of activity practised, so an in-depth study of the topic would be required.

## 5. Conclusions

The latent variables satisfaction and physical activities carried out at sports and health services aimed at adolescents are direct precursors of adherence and loyalty to the practice of physical activity in this age group. Human resources (sports instructors and service personnel) and physical activity are indirect precursors of loyalty via satisfaction. It is also important to note that service personnel play an important role in building loyalty among boys and girls at less consolidated stages.

## Figures and Tables

**Table 1:** Descriptive statistics, internal consistency, convergent and discriminant validity. Bivariate correlations.

Dimensions and Items	Mean	SD	Skewness	Kurtosis	Factor Loading		Correlation						
						1	2	3	4	5	6	7	8
1. Sports instructors; α = 0.84; AVE = 0.68; CR = 0.89						-	0.409 **	0.388 **	0.650 **	0.365 **	0.585 **	0.473 **	0.527 **
I am happy with the treatment I have received so far from the teacher/coach.	3.86	1.11	−0.814	0.060	0.818								
I believe that the teacher/coach has been paying appropriate attention to the users’ problems since day one.	3.80	1.04	−0.614	−0.228	0.846								
I believe that the teacher/coach adapts the classes/training to the customers’ interests/needs.	3.84	1.02	−0.627	−0.147	0.819								
I believe that the teacher/coach is encouraging the group sufficiently.	3.96	1.04	−0.776	−0.074	0.815								
2. Sports facilities; α = 0.85; AVE = 0.77; CR = 0.91						-	-	0.604 **	0.452 **	0.440 **	0.515 **	0.464 **	0.423 **
The changing rooms are sufficiently clean.	3.39	1.18	−0.278	−0.699	0.884								
The changing rooms are spacious enough.	3.45	1.11	−0.211	−0.701	0.881								
The facilities are clean enough.	3.44	1.14	−0.283	−0.652	0.869								
3. Equipment; α = 0.83; AVE = 0.75; CR = 0.90						-	-	-	0.491 **	0.439 **	0.527 **	0.417 **	0.411 **
There is sufficient equipment for training.	3.50	1.13	−0.333	−0.650	0.863								
The equipment is in good condition for use.	3.57	1.08	−0.333	−0.523	0.905								
The equipment is modern.	3.43	1.15	−0.293	−0.672	0.837								
4. Activity; α = 0.82; AVE = 0.58; CR = 0.87						-	-	-	-	0.499 **	0.659 **	0.563 **	0.619 **
The activity is enjoyable.	3.82	1.05	−0.610	−0.162	0.782								
The tasks carried out in the training sessions are diverse enough.	3.88	0.98	−0.617	−0.133	0.819								
Activities end at the appointed time.	3.85	1.10	−0.725	−0.219	0.723								
I get the expected results with this activity.	3.87	1.03	−0.769	0.145	0.783								
It was easy for me to join the activity I am participating in.	3.85	1.09	−0.745	−0.109	0.717								
5. Communication; α = 0.81; AVE = 0.73; CR = 0.89						-	-	-	-	-	0.542 **	0.555 **	0.510 **
The facilities have several means of receiving suggestions (suggestion box, bulletin board).	3.26	1.21	−0.164	−0.849	0.823								
The information on the activities offered at the centre is appropriate.	3.59	1.06	−0.304	−0.488	0.869								
The range of activities is constantly being updated.	3.55	1.09	−0.291	−0.590	0.871								
6. Service personnel; α = 0.80; AVE = 0.83; CR = 0.91						-	-	-	-	-	-	0.619 **	0.624 **
The staff are friendly.	3.80	1.06	−0.647	−0.094	0.916								
The staff at the facility have a good relationship with each other.	3.87	1.02	−0.602	−0.260	0.916								
7. Satisfaction; α = 0.89; AVE = 0.75; CR = 0.92						-	-	-	-	-	-	-	0.614 **
Joining this club was a good decision.	3.98	1.01	−0.786	0.096	0.871								
I am glad I joined this club.	4.05	0.95	−0.731	−0.080	0.894								
It was a good decision to engage in sports activities in this club.	4.04	0.95	−0.754	0.017	0.887								
I am pleased to be enrolled in this club.	4.06	0.98	−0.853	0.113	0.824								
8. Loyalty; α = 0.88; AVE = 0.70; CR = 0.92						-	-	-	-	-	-	-	-
I will share the positive aspects of this sports club with other people.	5.50	1.41	−1.004	0.778	0.855								
I will recommend this sports centre to anyone seeking my advice.	5.54	1.34	−0.850	0.190	0.892								
I will encourage my friends and family to participate in sports activities at this centre.	5.59	1.39	−0.897	0.158	0.860								
I would consider this club as my first choice for any sports service I might need.	5.50	1.45	−0.938	0.404	0.844								
In the next few years, I will take part in more sports activities in this club.	5.26	1.65	−0.882	0.050	0.734								

SD: Standard Deviation; AVE: Average Variance Extracted; CR: Composite Reliability; ** *p* < 0.001.

**Table 2:** Stages of change, gender, age, perceived quality, satisfaction, and loyalty. Percentages, means, and standard deviations. Contingency table and ANOVA.

Socio-Demographic Variables and Dimensions	Stages of Change						Groups			
	1	2	3	4	5	Total	Less Consolidated Stages	Later Stages	Comparison	Size Effect
Total	1.6%	8.7%	6.3%	14.1%	69.3%		16.6%	83.4%	0.009	
Male	1.8%	10.3%	6.8%	15.3%	65.9%		18.9%	81.1%		0.063
Female	1.3%	7.0%	5.9%	12.9%	73.0%		14.2%	85.8%		
Age	13.85 ± 1.29	14.04 ± 1.24	13.66 ± 1.26	13.79 ± 1.32	13.82 ± 1.33	13.83 ± 1.32	13.88 ± 1.26	13.82 ± 1.33	0.498	
INS	3.52 ± 1.17	3.65 ± 0.79	3.68 ± 0.95	3.69 ± 0.81	3.95 ± 0.86	3.86 ± 0.87	3.65 ± 0.89	3.90 ± 0.86	0.000	0.012
SP	3.64 ± 1.17	3.63 ± 0.75	3.49 ± 0.91	3.68 ± 0.84	3.92 ± 0.83	3.83 ± 0.84	3.58 ± 0.86	3.88 ± 0.83	0.000	0.017
SF	3.23 ± 1.19	3.33 ± 0.91	3.04 ± 1.09	3.36 ± 0.94	3.50 ± 1.01	3.43 ± 1.01	3.21 ± 1.02	3.47 ± 1.00	0.000	0.009
EQP	3.25 ± 1.21	3.43 ± 0.86	3.11 ± 1.11	3.46 ± 0.92	3.56 ± 0.97	3.50 ± 0.97	3.20 ± 1.00	3.54 ± 0.96	0.000	0.006
ACT	3.47 ± 1.10	3.62 ± 0.74	3.69 ± 0.85	3.74 ± 0.74	3.93 ± 0.79	3.86 ± 0.80	3.63 ± 0.82	3.90 ± 0.79	0.000	0.015
COMM	3.23 ± 1.12	3.38 ± 0.84	3.12 ± 1.03	3.30 ± 0.93	3.54 ± −95	3.46 ± 0.96	3.27 ± 0.95	3.50 ± 0.95	0.000	0.008
SATS	3.67 ± 1.22	3.70 ± 0.94	3.71 ± 1.01	3.79 ± 0.89	3.99 ± 0.90	3.91 ± 0.92	3.70 ± 0.99	3.95 ± 0.90	0.000	0.010
LOY	4.80 ± 1.61	5.13 ± 1.18	5.10 ± 1.28	5.28 ± 1.13	5.61 ± 1.18	5.48 ± 1.21	5.09 ± 1.26	5.56 ± 0.1.18	0.000	0.021

INS: Sports instructor; SP: Service personnel; SF: Sports facilities; EQP: Equipment; ACT: Activity; COMM: Communication; SATS: Satisfaction; LOY: Loyalty; 1: Precontemplation; 2: Contemplation; 3: Preparation; 4: Action; 5: Maintenance.

**Table 3:** Adjustment statistics for the models. Comparison between models using model 1 as correct in the two groups of users aged 12–16.

**Goodness-of-Fit Indices and Model Comparisons for Tested Models**							
**Model**	**CMIN**	**DF**	**CMIN/DF**	**CFI**	**RMSEA**	**ECVI**	**AIC**
0	896.370	349	2.568	0.965	0.041	1.165	1068.370
0a	633.172	349	1.814	0.922	0.059	3.426	805.172
0b	647.129	349	1.854	0.973	0.037	1.296	819.129
1	2231.567	698	3.197	0.948	0.036	1.502	2575.567
2	2270.842	719	3.158	0.948	0.035	1.500	2572.842
3	2282.322	732	3.118	0.948	0.035	1.492	2558.322
4	2333.449	753	3.099	0.947	0.035	1.497	2567.449
5	2344.171	755	3.105	0.947	0.035	1.501	2574.171
**Comparisons of Conditions Using Measurement Invariance Procedures**							
Model correct		**Model**	**Dif. DF**	**Dif. CMIN**	***p***	**Dif. CFI**	**Dif. RMSEA**
Assuming model 1 to be correct		2	21	39.275	0.009	0.000	−0.001
		3	34	50.756	0.032	0.000	−0.001
		4	55	101.882	0.000	−0.001	−0.001
		5	57	112.604	0.000	−0.001	−0.001
Assuming model 2 to be correct		3	13	11.481	0.571	0.000	0.000
		4	34	62.607	0.002	−0.001	0.000
		5	36	73.330	0.000	−0.001	0.000
Assuming model 3 to be correct		4	21	51.126	0.000	−0.001	0.000
		5	23	61.849	0.000	−0.001	0.000
Assuming model 4 to be correct		5	2	10.722	0.005	0.000	0.000

Note. Model 0, total number of minors; model 0a, minors at less consolidated stages; model 0b, minors at advanced stages; model 1, no parameters constrained to be equal across groups; model 2, factor loadings constrained to be equal; model 3, observed structural weights and factor loadings constrained to be equal; model 4, observed structural covariances, structural weights, and factor loadings constrained to be equal; model 5, observed structural residuals, structural covariances, structural weights, and factor loadings constrained to be equal. Dif. CMIN, difference between models; Dif. DF, difference between models; *p* = significance level between models; Dif. CFI, difference between models; Dif. RMSEA, difference between models.

**Table 4:** Comparison between standardised and unstandardised regression coefficients and significance levels of the two groups of users aged 12–16.

			Total Users		Less Consolidated Group		More Advanced Group	
			Direct Effects	Indirect Effects	Direct Effects	Indirect Effects	Direct Effects	Indirect Effects
Relations between Dimensions			Beta	Beta	Beta	Beta	Beta	Beta
SATS	←	INS	0.110 *	--	0.177	--	0.099 *	--
SATS	←	SS	0.447 **	--	0.431 **	--	0.446 **	--
SATS	←	COMM	0.015	--	0.078	--	0.003	--
SATS	←	ACT	0.306 **	--	0.299 *	--	0.307 **	--
SATS	←	EQP	0.024	--	0.024	--	0.017	--
SATS	←	SF	0.016	--	0.043	--	0.018	--
LOY	←	SATS	0.426 **	--	0.254	--	0.444 **	--
LOY	←	INS	0.057	0.047 *	0.091	--	0.051	0.044 *
LOY	←	SP	0.110	0.191 **	0.393 *	--	0.058	0.198 **
LOY	←	COMM	0.090	--	0.019	--	0.106 *	--
LOY	←	ACT	0.221 *	0.130 *	0.181	--	0.227 **	0.137 **
LOY	←	EQP	−0.045	--	0.055	--	−0.048	--
LOY	←	SF	0.040	--	−0.056	--	0.052	--

INS: Sports instructor; SP: Service personnel; SF: Sports facilities; EQP: Equipment; ACT: Activity; COMM: Communication; SATS: Satisfaction; LOY: Loyalty; * *p* < 0.05; ** *p* < 0.001.

## Data Availability

The data presented in this study are available on request from the corresponding authors.

## Abbreviations

TTM: Transtheoretical Model; DST: Self-determination Theory; BNP: Basic Psychological Needs; EPOD2: Sports Organisations Perception Scale, version 2; AVE: Average Variance Extracted; CR: Composite Reliability; SPSS: Statistical Package for the Social Sciences; AMOS: Analysis of Moment Structure; RMSEA: Root Mean Square Error of Approximation; CFI: Comparative Fix Index; ECVI: Expected Cross Validation Index; AIC: Akaike Information Criterion; CMIN: chi-squared; DF: Degrees of Freedom; Δ: Increase.
